# Grating based interferometric devices on a silicon photonic platform

**DOI:** 10.1038/s41598-026-56188-z

**Published:** 2026-06-13

**Authors:** Thomas J. G. Mikhail, Seif M. Swillam, Mohamed A. Swillam

**Affiliations:** Department of Physics, School of Sciences and Engineering, American University in Cairo, AUC Avenue, New Cairo, 74, 11835 Egypt

**Keywords:** Optics and photonics, Physics

## Abstract

This work presents a class of integrated photonic interferometers based on the Michelson architecture, implemented on a silicon-on-insulator platform operating at 1.5–1.6 $\mu$m. The devices combine directional couplers, subwavelength gratings (SWG), and Bragg grating reflectors. Furthermore, by cascading and branching multiple Michelson units, we demonstrate more complex interferometric structures for specialized applications. The integration of SWG sections enables fine-tuning of the effective refractive index and enhances sensitivity for sensing applications through increased evanescent field interaction with the surrounding medium. Selected designs are fabricated and experimentally characterized. Fabricated Fabry-Pérot devices achieve quality factors exceeding 6,500, while Michelson-based structures exhibit extinction ratios up to 43 dB, demonstrating good qualitative agreement with simulations. Finally, we explore potential applications optical filtering, sensing, and electro-optic modulation.

## Introduction

Over the past two decades, silicon-on-insulator (SOI) has become one of the most widely adopted platforms in integrated photonics, largely benefiting from the fabrication infrastructure developed for the complementary metal-oxide-semiconductor (CMOS) electronics industry^1,2^. Its high refractive index contrast enables tight optical confinement, allowing for submicron waveguide geometries, dense component integration, and low loss at the infrared wavelengths commonly used in optical data transmission (1300–1550 nm). The SOI platform supports a wide range of passive and active photonic components, and offers significant advantages over free-space or fiber-based technologies in terms of cost, complexity, speed, and energy efficiency. These components can be integrated into complex photonic circuits for applications such as optical communications, signal processing, sensing, and spectroscopy, supporting scalable, high-volume production through standard semiconductor processes.

In modern photonic circuits, minimizing the device footprint while maintaining performance is a key design priority^3,4^. Compactness not only enables higher integration density but also reduces propagation losses and improves fabrication yield. Interferometric structures are widely used for filtering, modulation, sensing, and delay-line applications. Numerous approaches have been explored to reduce the footprint of such devices without compromising optical performance, including the use of folded or looped optical paths, resonant cavities, and subwavelength structures to enhance phase control in compact regions^5–8^. Reflective configurations, in particular, allow effective use of on-chip space by folding the optical path length, enabling resonant or interferometric behavior within a smaller footprint.

Subwavelength grating (SWG) waveguides and engineered slow-light structures offer versatile control over the effective index, enabling further design flexibility or extending phase differences in devices without relying on footprint heavy optical path differences^9,10^. Furthermore, in the context of sensing, enhanced interaction between the guided mode and the surrounding environment leads to improved sensitivity. Recent advancements in integrated photonic meta-waveguides have demonstrated more complex, multifunctional control over mode, phase, and dispersion^11^, and this is a rich and evolving field. However, for the interferometric architectures proposed within this paper, the primary requirement is precise phase accumulation, a compact footprint, and strong environmental mode overlap. For these specific objectives, standard 1D SWG structures are sufficient and avoid the increased fabrication complexity and potential scattering losses often associated with more intricate 2D metamaterial designs.

In this work, we design, fabricate, and compare several compact interferometric devices implemented on the silicon-on-insulator (SOI) platform. Existing reflective interferometric designs often suffer from high spatial overhead or rely on specialized, non-standard components. For example, conventional Bragg reflectors in similar cavities^12^ can occupy the vast majority of the device footprint, which inherently complicates fine phase control and restricts active tuning capabilities. The proposed architectures resolve this through the incorporation of circular Bragg grating (CBG) mirrors. The integration of CBGs in this context yields a reflector footprint of 4.49 $\mu$m $\times$ 4.54 $\mu$, providing a quantifiable reduction in spatial overhead compared to conventional designs, enabling much denser device integration. The devices are based on a shared architecture that combines directional couplers, Bragg reflectors, and subwavelength grating waveguides. However, the basic architecture is compatible with other components depending on application. Within these configurations, SWG structures are utilized specifically to maximize the phase difference per unit area, reducing the physical length required for targeted optical transformations, and to maximize sensitivity in sensing applications. All devices operate in the 1.5–1.6 $\mu$m wavelength range and are investigated for applications in filtering, modulation, and sensing. However, the large operating bandwidth of the reflector means these devices can be utilized in other bands as well. We focus on two canonical interferometric configurations, the Michelson interferometer and the Fabry-Pérot resonator. Notably, the Fabry-Pérot resonator does not suffer from the FSR — quality factor tradeoff seen in microring resonators by using a straight cavity configuration, avoiding radiative losses.

Building upon these basic structures, we explore design variants for more specific applications. Our approach extends these structural improvements into novel configurations. The Michelson interferometer is extended in two ways: first, by cascading the original Michelson design with an additional directional coupler to form a grating-terminated Mach-Zehnder interferometer (GT-MZI), and second, by branching the arms to form a compact quadrature modulator (IQM) with a folded layout. All devices are fabricated using electron beam lithography (EBL) and characterized. Furthermore, we discuss ways to expand the device for other applications, including sensing, modulation and filtering.

## Simulation and device modeling

**Fig. 1:**
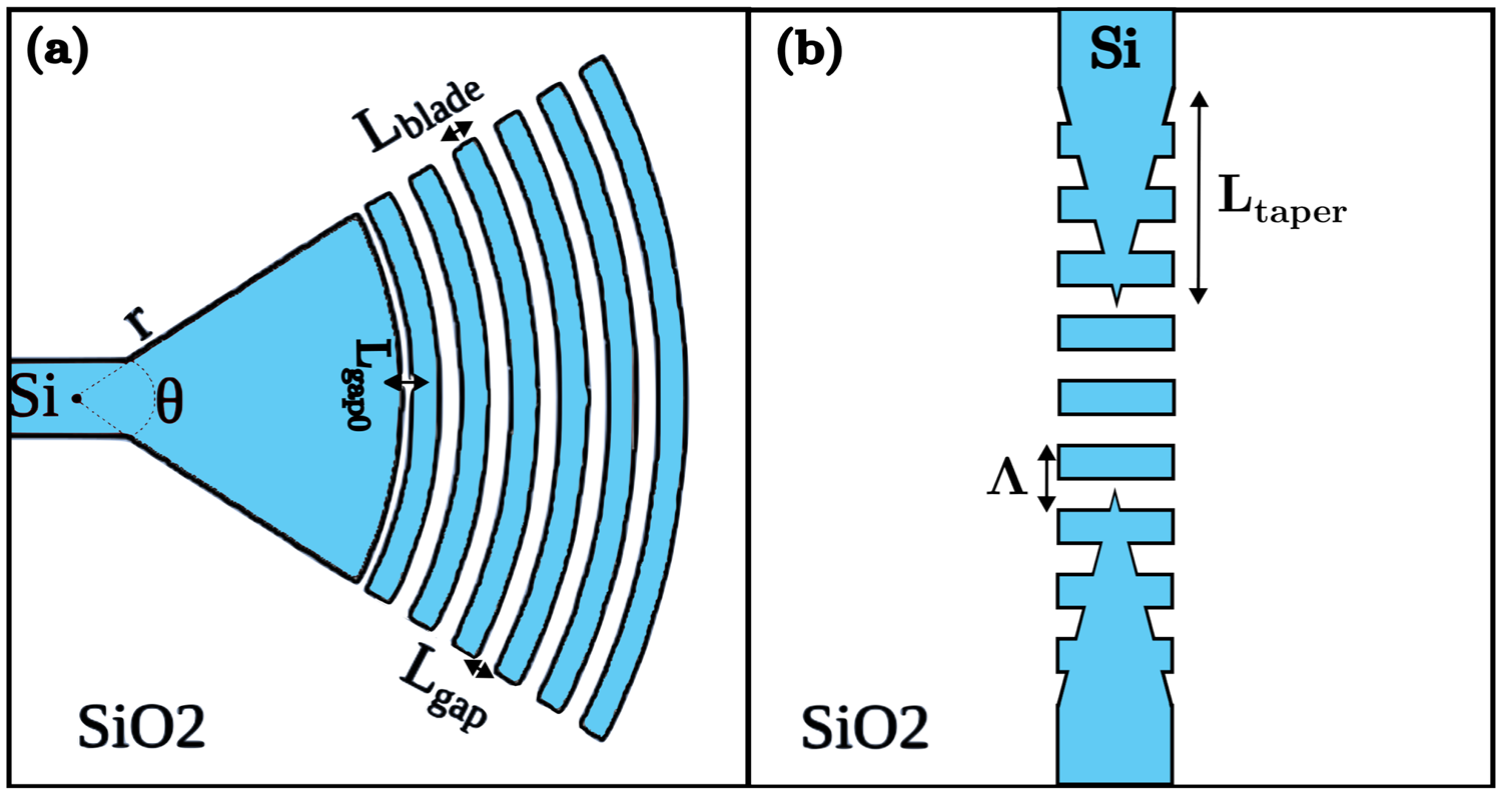
Main design parameters for the devices used in this study: (**a**) a circular Bragg Grating Reflector and (**b**) Sub-wavelength gratings.

**Fig. 2:**
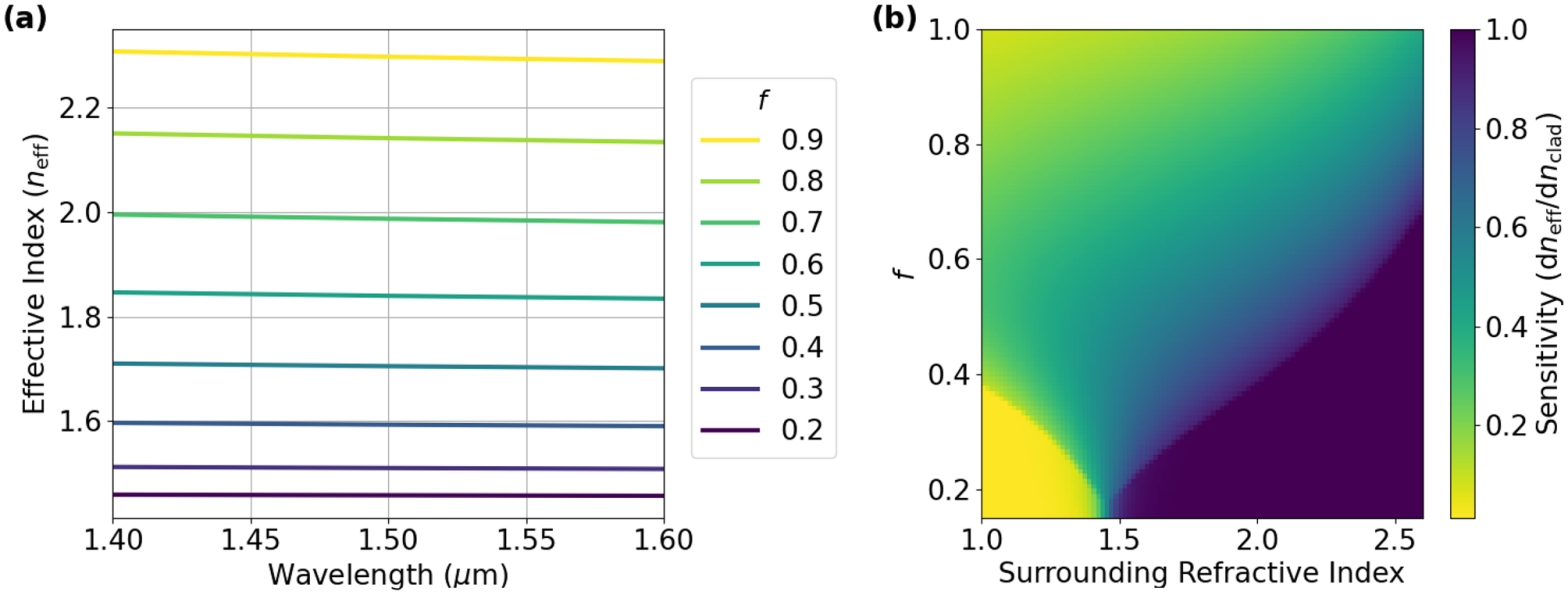
(**a**) Effective index of the fundamental TE mode for subwavelength grating (SWG) waveguides on the SOI platform with different duty cycles as a function of wavelength, calculated using 1 and a mode solver (**b**) Sensitivity of a 220$\times$500 nm SOI SWG waveguide’s fundamental TE mode ($\partial n_{\text {eff,mode}}/\partial n_{\text {surrounding}}$) as a function of duty cycle and surrounding refractive index. Lower duty cycles increase the SWG material’s intrinsic dependence on the surrounding medium (via Equation1), leading to higher sensitivity in principle. However, the actual waveguide mode sensitivity also depends on optical confinement: at low surrounding index (e.g., air), high index contrast produces strong mode confinement that partially suppresses this sensitivity, while at high surrounding index, reduced contrast leads to weak confinement and sensitivity approaching unity. These high-sensitivity regions (low duty cycle combined with high surrounding index) may correspond to weakly guided or leaky modes and may not be physically feasible.

Numerical simulations were performed using a combination of three-dimensional finite-difference time-domain (FDTD), and an S-parameter based photonic circuit solver, implemented in the Ansys Optics software suite^13^, and an open-source FEM eigenmode solver^14^. Material properties for silicon and silicon dioxide (SOI platform) were taken from Palik^15^, and all devices were analyzed over the wavelength range of 1.5–1.6 $\mu$m. Waveguide effective indices and field distributions were computed using the mode solver.

A subwavelength grating is a periodic dielectric structure with a period ($\Lambda$) smaller than the wavelength of the incident or propagating light, as shown in Fig. 1. This dimensional constraint suppresses diffraction into higher orders, causing the structure to behave as a homogenous material with a tailorable effective refractive index. For subwavelength grating structures, the grating region can be approximated as a homogeneous medium with effective index given by the Rytov formula:

1 $$\begin{aligned} n_{\textrm{eq}}^{2} = f\,n_{\textrm{core}}^{2} + (1-f)\,n_{\textrm{bg}}^{2}, \end{aligned}$$

where $f = a/\Lambda$ is the duty cycle, or fill factor, with *a* representing the length of the core material segment, and $n_{\textrm{core}}$ and $n_{\textrm{bg}}$ the refractive indices of the core and background materials, respectively. Notably, this approximation requires the grating period to be smaller than the guided wavelength ($\Lambda \ll \lambda / n_{\textrm{eff}}$). Coupling light between a continuous waveguide and a subwavelength grating requires a taper to minimize mode mismatch and scattering. The length of this taper ($L_{taper}$) simply must be sufficient to ensure an adiabatic mode transition, preventing insertion loss and reflections.The devices in this work utilize a period of $\Lambda = 240$ nm. While this value approaches the limits of the strict subwavelength condition at the 1.55 $\mu$m operating wavelength, the Rytov model maintains sufficient accuracy for effective medium estimation. Prior studies have demonstrated that applying this approximation to comparable grating periods yields theoretical models that agree well with experimental device performance^7^. The resulting equivalent index was then assigned to a slab waveguide, from which the fundamental TE mode was extracted. Figure 2a illustrates the variation of effective index with duty cycle for SWG waveguides of width 0.5 $\mu$m and height 0.22 $\mu$m. SWG sections were connected to strip waveguides through short taper transitions to minimize scattering losses and ensure adiabatic mode conversion between regions of different effective index.

From Eq. 1, the effective index of a SWG mode depends on both the properties of the SWG medium itself and the mode’s interaction with the surrounding material. Consequently, the sensitivity of the effective index to environmental changes is influenced by multiple factors and should be optimized for the specific application. For instance, the sensitivity (S) of a SWG waveguide depends on both the refractive index surrounding material or analyte and the duty cycle, as illustrated in Fig.2b for a standard 500$\times$220 nm SOI waveguide.

**Fig. 3:**
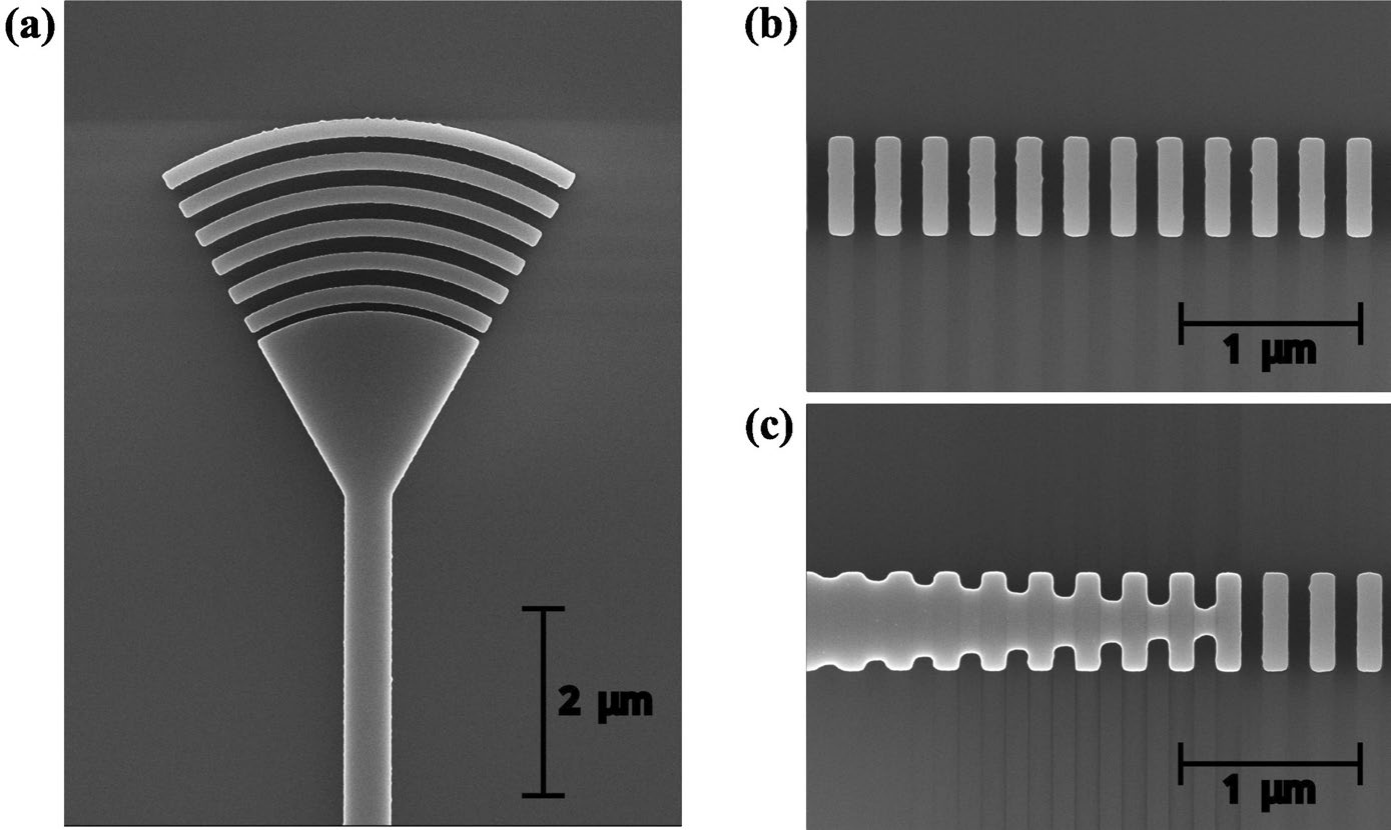
SEM images of fabricated components: (**a**) circular Bragg grating reflector, (**b**) a SWG waveguide section, (**c**) a SWG taper section, connecting to a standard 0.5 $\mu m$ waveguide.

While SWG waveguides effectively enhance the evanescent field for environmental interaction, this geometry increases the mode overlap with the vertical sidewalls of the silicon segments. Consequently, sidewall roughness, such as those observed in the fabricated structures in Fig. 3, introduces higher scattering losses compared to standard waveguides^16^. These scattering losses can be mitigated through mature commercial foundry processes or, for electron beam lithography, by minimizing the writing pitch to improve edge definition^7^. Furthermore, provided the SWG waveguide remains strictly single-mode, dimensional variations along the longitudinal direction of propagation do not induce significant performance degradation^16^. Established literature indicates that propagation losses for subwavelength grating waveguides on the SOI platform typically range from 2 to 5 dB/cm^8,10,17^.

The other major element used in this study is the compact circular Bragg grating (CBG) mirror first proposed in^18^. The circular Bragg grating functions as a broadband reflector. The architecture consists of a single-mode strip waveguide, a pie-shaped taper, and a series of concentric circular grating blades, as shown in 1a. Light propagating in the strip waveguide enters the FPR and undergoes Huygens-Fresnel diffraction. Because the waveguide aperture is small relative to the operational wavelength, the light diffracts into a circular wavefront. The taper expands this wave to align with the curvature of the subsequent circular grating blades, which reflect the light back into the input waveguide.

The reflection mechanism is governed by the first-order Bragg condition:

2 $$\begin{aligned} n_t L_{gap} + n_b L_{blade} = \frac{\lambda _0}{2} \end{aligned}$$

where $W_t$ and $W_b$ denote the widths of the grating trenches and blades, respectively. The variables $n_t$ and $n_b$ represent the effective refractive indices of the trenches and blades, and $\lambda _0$ is the center Bragg wavelength.

A 50% duty cycle ($W_t = W_b$) maximizes grating strength and reflection bandwidth^18^. The refractive index $n_t$ is determined by the cladding material (1.4447 for SiO_2_^15^), while $n_b$ is approximated using the effective index of a slab waveguide of equivalent thickness, which can be calculated with a eigenmode solver. For a target wavelength of $\lambda _0 = 1.55$ $\mu$m, this results in trench and blade widths of 180 nm.

**Fig. 4:**
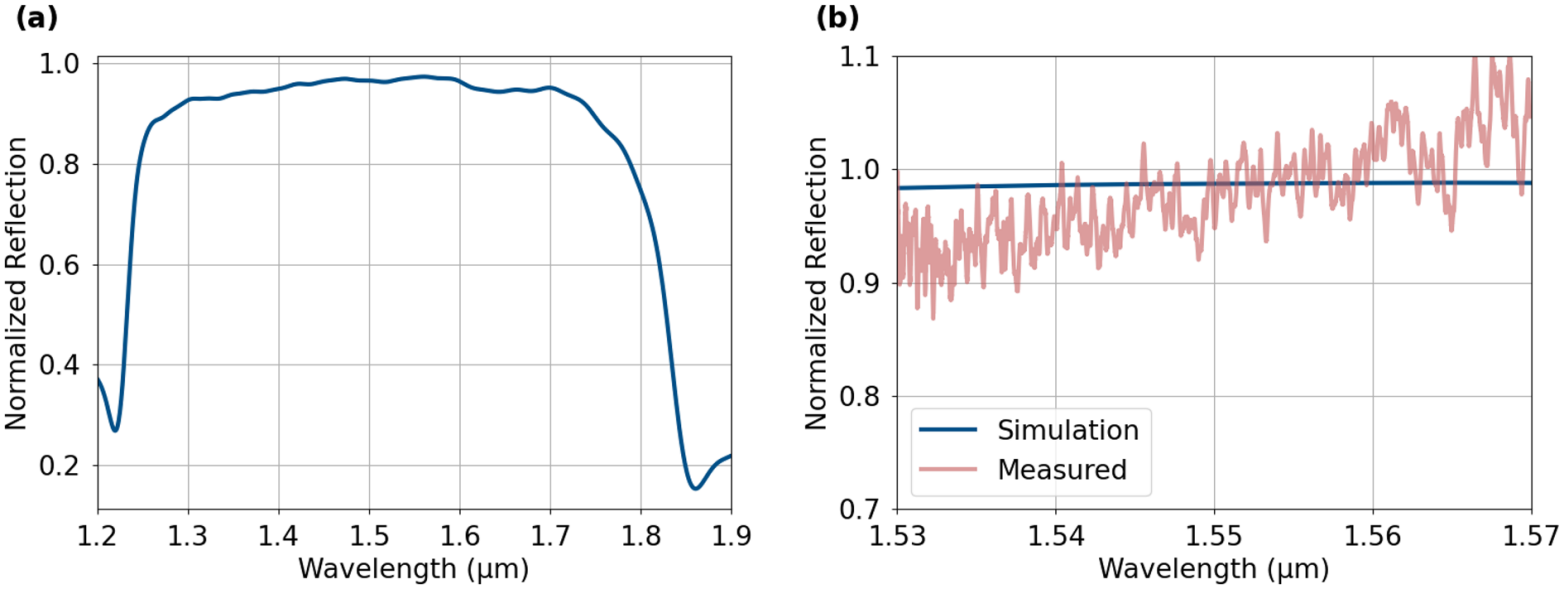
(**a**) Simulated reflection spectra for the integrated bragg grating reflector utilized across all devices over the wavelength range of 1.2–1.9$\mu m$ (**b**) Comparison of simulated and measured reflection spectra for the device between 1.53–1.57$\mu m$. The data acquired with the power tap has a low signal-to-noise ratio due to the low power differential between the reflection and transmission. Normalized transmission values exceeding 1.0 are the result of these measurement artifacts and do not indicate optical gain.

A taper arc angle ($\theta$) of $60^{\circ }$ is selected because it provides sufficient width to capture the majority of the diffracting wavefront’s optical power, avoiding the unnecessary footprint increases associated with larger angles. Furthermore, utilizing a smaller angle reduces the lateral expansion of the optical mode, which increases radiation leakage at the taper boundaries and proportionally reduces the overall reflectivity of the mirror, as well as require a larger radius. Geometrically, the incorporation of the taper with length *r* shifts the center of the grating curvature, setting the minimum gap size of the first grating blade ($L_{gap0}$). This removes the requirement for a difficult to fabricate gap between the waveguide and the first reflector. For the chosen radius of 2$\mu m$, ($L_{gap0}$) is 100 nm, which is well within fabrication tolerance. The structure utilizes six grating blades, which is sufficient for near total reflection, and a greater number of blades beyond this having little effect on performance^18^. In total,the footprint of 4.49 $\mu$m $\times$ 4.54 $\mu$m, and produces a reflectivity exceeding 90% over a 500 nm bandwidth (1263–1763 nm) and exceeding 95% over a 397 nm bandwidth (1340–1737 nm), as shown in Fig. 4.

This mirror provides several advantages over conventional Bragg gratings: it achieves broadband reflectivity exceeding 95% across a 397 nm range, while maintaining a much smaller footprint. In contrast, traditional Bragg gratings typically require many more periods to reach similar reflectivity, resulting in significantly larger device sizes and broader bandwidths^19^. The CBG design also exhibits high tolerance to fabrication imperfections, making it particularly suitable for integration.

The CBG reflector was simulated using 3D FDTD to obtain its reflective and phase responses over the 1.5–1.6 $\mu$m wavelength band, extracted as scattering parameters (S-parameters) for use in circuit-level modeling. To ensure numerical convergence, a non-uniform mesh with a high grid resolution applied across the high-index contrast boundaries of CBG structure. Perfectly matched layer (PML) boundary conditions were applied on all axes to absorb outward radiating waves and prevent artificial reflections within the computational domain. Figure 3 shows a SEM images for on the devices utilized in this work, and Fig. 4b shows the experimental reflection for this device relative to simulation.

These final S-parameter matrices were subsequently combined within the photonic circuit solver. This allowed for quickly evaluating the composite device architectures seen in the next section.

## Device architectures

**Fig. 5:**
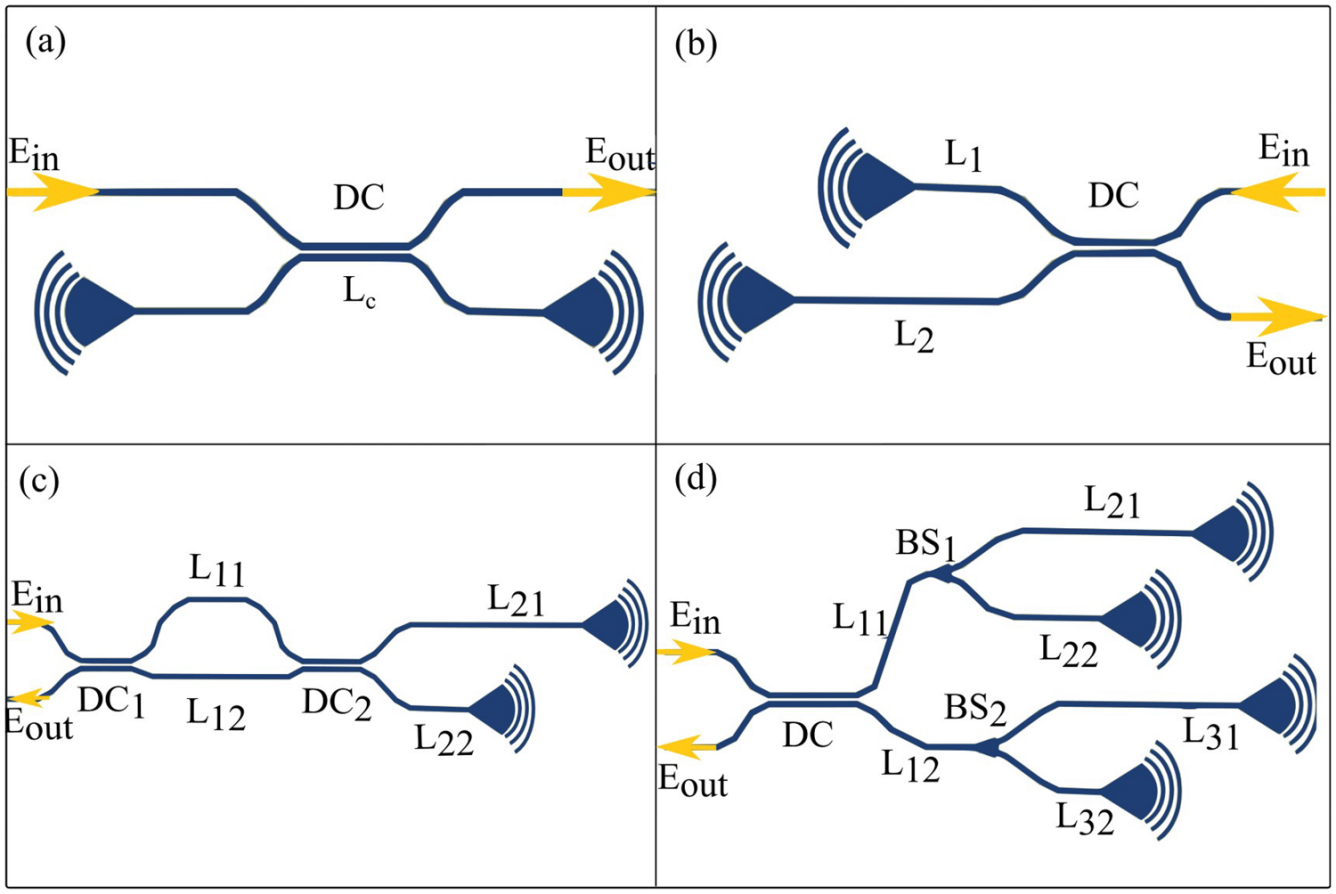
Proposed designs in this paper with labeled parameters. All interferometers are composed of directional couplers (DC) and Bragg grating reflectors with maximized reflectivity. (**a**) illustrates a transversely coupled Fabry-Pérot resonator, while (**b**) shows a Michelson interferometer. (**c**) The other two sub-Figures display more complex devices based on the Michelson design, with (**c**) cascading an additional DC to form a reflection terminated Mach-Zehnder interferometer and (**d**) branching the device with y -branches, forming a highly tunable modulator.

This section presents the proposed device architectures, with a summary of the devices and main design parameters shown in Fig. 5. Potential applications are explored, and simulation results are provided for the devices with varied parameters.

An inherent design consideration in these reflection-based architectures is the bidirectional behavior of standard directional couplers. This results in a portion of the reflected light being routed back toward the input port, a behavior consistent with their free-space equivalents. To prevent multipath noise and ensure laser stability, this back-reflection necessitates upstream optical isolation. During our device characterization, this was mitigated using a tunable laser source equipped with a built-in optical isolator, which ensured stable operation. While standard couplers are utilized here to demonstrate the baseline architecture and rely on standard external isolation for testing, fully monolithic integrated systems will eventually require on-chip isolation. Future iterations of these devices can be extended with asymmetric coupling structures, such as photonic crystals^20^ or magneto-optical waveguide couplers^21^, to suppress reverse propagation directly on the chip.

### Fabry-Pérot interferometer

The Fabry-Pérot Interferometer/Resonator (FP) was developed over a century ago by Charles Fabry and Alfred Pérot, has played a critical role in the advancement of optical science. It operates by allowing light to reflect back and forth between two partially reflective surfaces, producing wavelength dependent interference patterns. This property has made the FP a useful tool in applications such as spectroscopy, laser tuning, and optical communications^22,23^.

However, in integrated photonics, FPs are far less common than the more ubiquitous microring resonators, despite being able to achieve similar performance in terms of Q-factor^24^. Furthermore, ring resonators are not suitable for applications requiring large free spectral ranges (FSR), as these need small radii which increases radiation losses and can be difficult to fabricate. Furthermore,, the integration of SWG metamaterials requires a significant increase in the device footprint; in conventional strip waveguides, tight bending radii (R<5 $\mu m$) are possible due to strong modal confinement, but the periodic refractive index perturbation of an SWG structure induces substantially larger radiative bend losses. To suppress these losses and maintain a high-quality factor, the bending radius must be sufficiently large to ensure the optical mode remains adiabatically guided without significant leakage into the cladding^25^. This is also true of a range other geometries, such as slots and gratings.

Conversely, integrated FPs suffer from issues related to the difficulty in implementing reflectors on SOI, which requires either metal deposition for reflection, increase the contrast at the boundary of the device by exposing it to the air with an additional etching step, or large Bragg reflectors/loop mirrors with a size/bandwidth trade off. FP designs fall into two categories: more traditional designs composed of reflectors with an imperfect reflection coefficient, where the light enters the cavity and resonates within, and transversely coupled versions, where the cavity is composed of a length of waveguide terminated on each end with a reflector, and evanescent waveguide coupling is used to enter light into the cavity. In the first category, Sun et al^26^. used a Sagnac loop based FP resonator with phase tuning by PN junctions to create a tunable silicon comb filters with a Q-factor of 52,000, while Liu et al^27^. used two identical Bragg gratings as reflecting mirrors, with a straight waveguide in between, thereby acting as a cavity, achieving a maximum Q factor of 1514. There are also have been proposals for programmable devices, using microelectromechanical (MEMS) actuator tuned sagnac loop mirrors^28^ or thermo-optically tuned Bragg gratings^29^.

Transversely coupled FP interferometers also have had multiple proposed designs, as well as tunable designs, depending on the use of heaters to control the coupling coefficient and phase accumulation^12^. These can achieve high Q factors, such as Xu et al^30^., who created a design based on a multimode retro-reflector and achieved a Q factor of several million. However, this design suffers from being difficult to control due to it’s reliance on mode conversion in the cavity. Furthermore, designs based on Bragg gratings, such as by Saber et al.^12^, have large footprints, with the aforementioned design relying on reflectors 480 $\mu m$ long.

In integrated architectures, optical properties such as coupling ratio and resonance wavelength can be carefully controlled, while additional tuning mechanisms, such as thermal phase shifters or carrier-injection regions, provide further flexibility. As a result, the compact FP can be dynamically reconfigured and precisely engineered for a wide range of applications, including wavelength filtering, multiplexing in optical communication systems, integrated biosensors, and emerging technologies in quantum photonics^31^.

In this work, we propose a transversely coupled design utilizing circular Bragg grating reflectors, as shown in Fig. 5a. The main parameters of the device are the cavity arm length (*L*), the coupling ratio of the directional coupler (CR), and the reflectivity of the mirrors as a function of wavelength ($R(\lambda )$). Additionally, the effective index ($n_{eff}$) of the cavity arms plays a significant role in the device’s performance in the wavelength dependent phase term $\beta$, which is related to the index by the relation:

3 $$\begin{aligned} \beta (\lambda )=\frac{2\pi n_{eff}(\lambda )}{\lambda } \end{aligned}$$

Which relates to the device transmission response by:

4 $$\begin{aligned} T(\lambda ) = (1-\kappa )\, \frac{\big (1 - R(\lambda )^2\big )^2 \;+\; 4\,R(\lambda )^2\sin ^2\!\big (\beta (\lambda )L_c\,\big )}{\big (1 - R(\lambda )^2(1-\kappa )\big )^2 \;+\; 4\,R(\lambda )^2(1-\kappa )\,\sin ^2\!\big (\beta (\lambda )\,L_c\big )} \end{aligned}$$

This means the device resonates at $\beta L=m\pi$, resulting in a resonance condition similar to its classical inspiration:

5 $$\begin{aligned} n_{\text {eff}}L_c = m\lambda _{\text {res}} \end{aligned}$$

For spacing between resonances, or the free spectral range (FSR), dispersion must be taken into effect, so it is more accurate to use the group index $n_g$:

6 $$\begin{aligned} \text {FSR} = \frac{\lambda _{\text {res}}^2}{2n_g L_c} \end{aligned}$$

Finesse(F), on the other hand,describes the sharpness of the resonances, and is given by:

7 $$\begin{aligned} F = \frac{\pi \sqrt{R}(1-\kappa )}{1-R(1-\kappa )} \end{aligned}$$

This is inversely related to the full width at half-maximum of the resonances (FWHM):

8 $$\begin{aligned} \text {FWHM}=\frac{\lambda _{res}^2}{n_gL_cF} \end{aligned}$$

Finally, the quality factor (Q-factor) indicates how long light remains trapped in the cavity, making it an important Figure of merit (FOM) in optical resonators. It can be defined as the ratio between the resonance wavelength and the FWHM:

9 $$\begin{aligned} Q = \frac{\lambda _{res}}{\text {FWHM}} \end{aligned}$$

Equivalent to:

10 $$\begin{aligned} Q = \frac{ n_{g}L_c\pi \sqrt{R}(1-\kappa )}{\lambda _{res}(1-R(1-\kappa ))} \end{aligned}$$

A higher Q-factor indicates narrower resonances and longer photon lifetimes. This is important for many applications, such as optical signal processing, where sharp spectral filtering and precise control of pulse delay are often required, and in sensing, where they allow for substantially higher light-matter interactions, increasing sensitivity. For this reason, Q-factors are widely used as a benchmark parameter when comparing optical resonators. From Eq. 9, it follows that the quality factor is inversely proportionate to $\kappa$ and proportionate to *R*, $n_g$ and $L_c$. A constraint of the proposed FP architecture is the maximum reflectivity of the CBG mirrors ($R \approx 0.95$), which restricts the cavity round-trip survival probability to $R^2 \approx 0.90$.

**Fig. 6:**
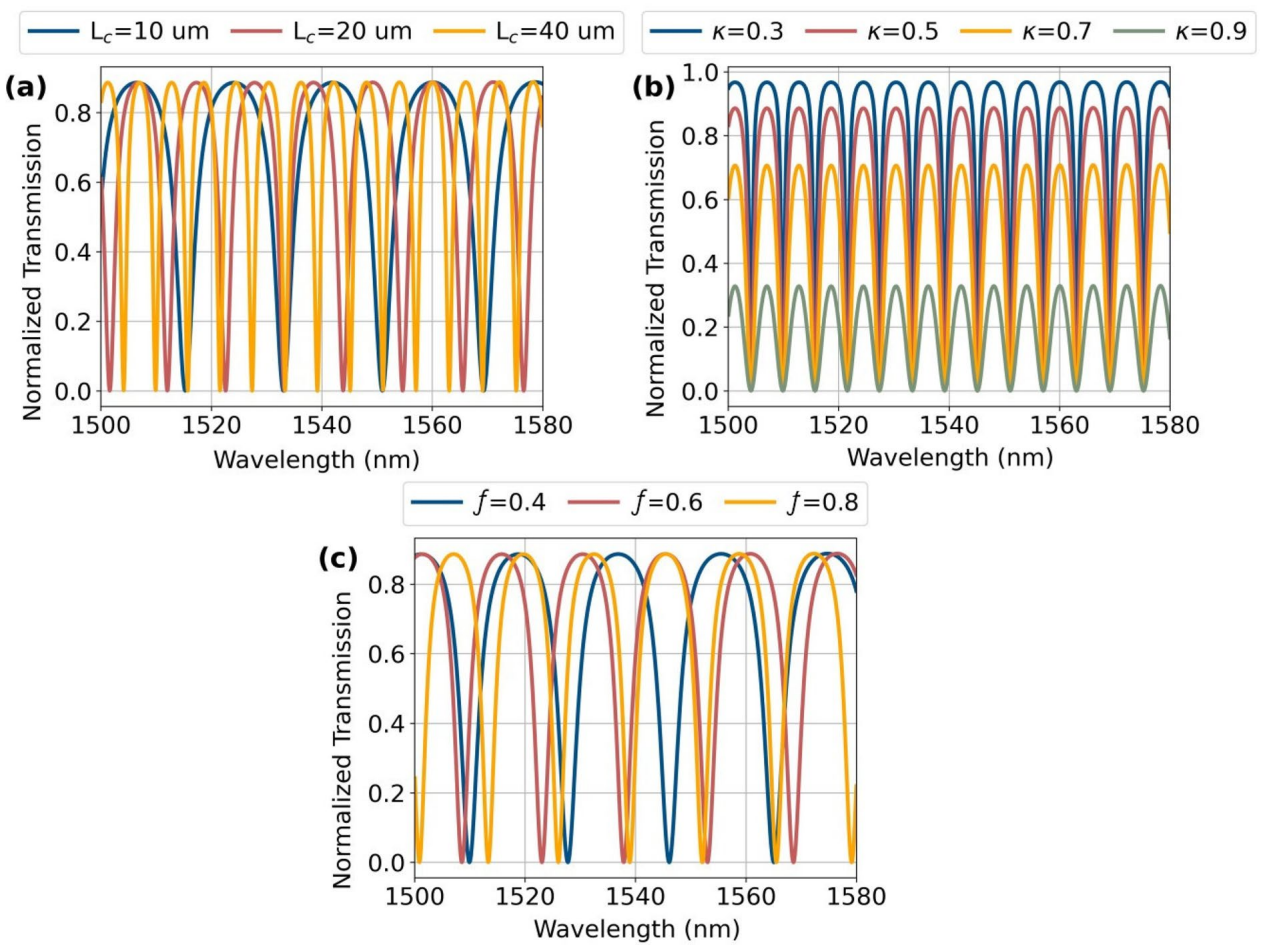
Effect of varying (**a**) cavity path lengths in an FP with $\kappa =0.5$ (**b**) $\kappa =0.5$ with L$_c=20$ $\mu$m (**c**) replacing the waveguides of cavity with different duty cycles $f$ of SWG.

Because the resonant properties depend directly on the cavity length, mirror reflectivity, coupling ratio, and effective index, careful tuning of these parameters allows the device to be tailored for specific functionalities. Figures 6 demonstrates the effect of changing design parameters on the output spectrum between 1.5–1.58 $\mu$m.

This sensitivity to changes in the arm’s $n_{eff}$ allows the device to have potential applications in sensing or optical modulation. For modulation, the depth of modulation can be controlled by changing the coupling ratio into the resonant cavity, while the resonant wavelengths can be modified by altering the $n_{eff}$ of the cavity through heaters or electro-optic tuning. Similarly, sensing can be achieved by exposing the cavity to the environment, altering its $n_{eff}$. This is especially true in the case of the SWG assisted designs, where the effective index of the waveguide matches the surrounding analyte more closely. Potential sensing applications are demonstrated in Section Discussion. By utilizing a straight Fabry-Pérot cavity, the proposed design entirely bypasses the aforementioned SWG bending-induced scattering losses, allowing for the direct integration of standard rectangular SWGs to maximize sensitivity without compromising the resonator’s structural simplicity^32^.

Furthermore, the input port of the device can function similarly to a drop ports. Drop ports are frequently used in MRR for applications such as wavelength-division multiplexing (WDM)^33–35^, channel routing^36,37^, and signal monitoring. By engineering the coupling regions, the drop port can be designed to extract a specific wavelength channel while leaving the remaining spectrum to continue propagating. This configuration improves the versatility of the device, and can also enable compact integration of complex filtering and switching functionalities within photonic circuits. Section Discussion explores this as a potential application.

Compared to MRRs, the proposed FP architecture presents a distinct set of operational trade-offs and structural capabilities. While MRRs can achieve ultra-high Q-factors, the FP’s Q-factor is inherently constrained by the maximum reflectivity of the CBG mirrors, which limits the round-trip survival probability. However, as previously mentioned, achieving a large FSR in MRRs necessitates small bend radii, increasing radiation losses. This limitation is amplified when SWGs or slot waveguides, as the periodic index perturbations induce substantial radiative bend losses. To mitigate this in MRRs, the bend radius must be increased, thereby enlarging the device footprint and drastically reducing the FSR. The proposed straight-cavity FP architecture bypasses these bending-induced losses entirely, allowing for the direct integration of SWGs, slots, or other structures for specific applications within a compact footprint. Ultimately, while being unable to match theoretical maximums for MRR in terms of ultra-high-Q applications, this FP architecture provides a specialized alternative for designs requiring SWG integration, extended FSRs, and compact routing capabilities.

### Michelson interferometer

The second proposed device is a Michelson interferometer (MI) design based on the same set of components. Originally introduced in the late 19th century, is another fundamental device in optical science. It operates by splitting light into two arms, reflecting it back with mirrors, and recombining the beams to generate interference patterns determined by the optical path difference. This direct sensitivity to phase and medium makes the MI useful for precision measurements and control in spectroscopy, and dimensional metrology^38^.

Unlike the Fabry-Pérot, which relies on multiple round-trip reflections between two mirrors to create a cavity, the MI directly translates changes in optical path difference into measurable interference fringes, which are dependent on phase, optical path length, or refractive index variations. The architecture can also be extended through the integration of active tuning mechanisms, such as thermo-optic phase shifters or carrier injection, which allow reconfigurable control over the interference condition.

Similar to the Fabry-Pérot, reflectors are usually achieved with Sagnac loop mirrors or Bragg grating reflectors, and are far less common than another commonly integrated interferometer, the Mach-Zehnder interferometer. Similarly, they face the same issues with these reflectors; the limited bandwidth of loop reflectors and the size/bandwidth trade-off of Bragg grating reflectors. Gerguis and Qi^39^ developed a Michelson interferometer for sensing based on a slotted Bragg reflector. More complex designs have also been attempted, such as a design based on multi-material, spiral Bragg gratings by Ghoname et al^40^, designed for optical modulation. Similarly, Patel et al.^41^and Xu et al^42^. developed active MI modulators terminated by loop mirrors. There has also been use as a low power switch, as demonstrated by Lu et al.^43^.

The design proposed in this paper, shown in Fig. 5b, is based on a DC to split the input light and recombine it after being reflected at the end of the two interferometer arms. Each arm has the light propagate through it twice, resulting in an accumulated phase difference between the two arms of:

11 $$\begin{aligned} \Delta \phi = (\frac{4\pi }{\lambda })(n_2L_2-n_1L_1) \end{aligned}$$

Essentially double that of a traditional MZI. For an MI with a DC coupling ratio $\kappa$, this results in a spectral output of:

12 $$\begin{aligned} T = {(1-2\kappa )}^2+ 4\kappa (1-\kappa )\cos ^2(\Delta \phi ) \end{aligned}$$

With resonances occurring for integers m satisfying:

13 $$\begin{aligned} \lambda _{res}=\frac{2(n_2L_2-n_1L_1)}{m} \end{aligned}$$

As such, the FSR is given by

14 $$\begin{aligned} FSR=\frac{\lambda ^2}{2(n_{g2}L_2-n_{g1}L_1)} \end{aligned}$$

**Fig. 7:**
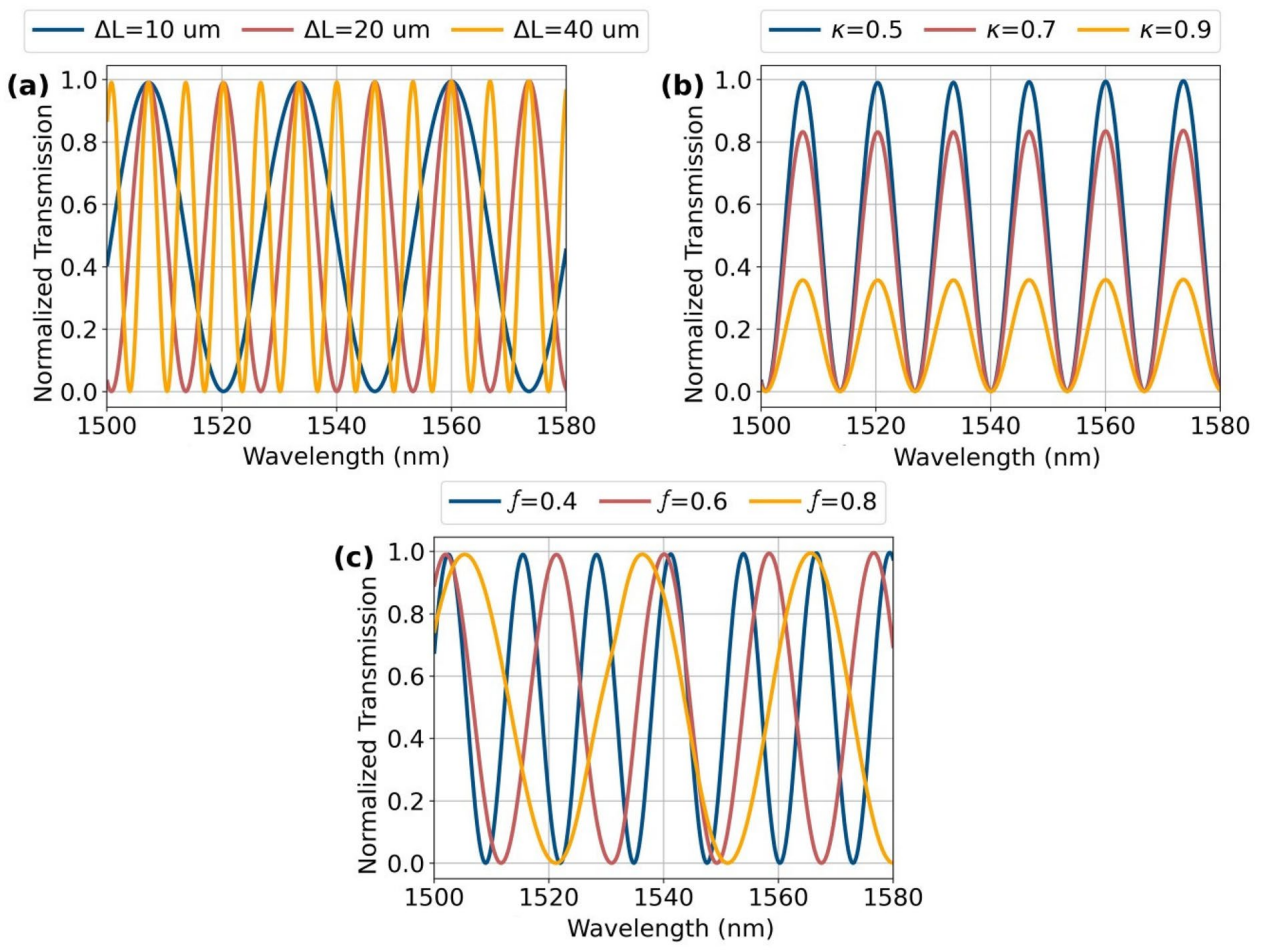
Effect of varying (**a**) path length differences in an MI with $\kappa =0.5$ (**b**) $\kappa =0.5$ with $\Delta$L$=20 \mu$m (**c**) replacing the waveguides on one arm with different duty cycles $f$ of SWG in michelson with equal arm lengths of L=40$\mu$m.

The performance of the Michelson interferometer is primarily determined by the coupling ratio, the optical path difference, and the reflector properties, enabling precise tailoring for a range of applications. As demonstrated earlier for Fabry-Pérot cavities, systematic variation of these parameters produces predictable changes in both the interference spectrum and device sensitivity. Figure 7 shows how varying these parameters affects output of the device.

Dynamic tuning of the interferometer can be achieved through integrated heaters or electro-optic modulation, which alter the effective index of the arms. This functionality enables active stabilization, wavelength tuning, and compensation for environmental drift. The twofold thermal sensitivity of the Michelson configuration further supports compact device footprints and enhanced modulation efficiency compared to Mach-Zehnder designs.

### Combined interferometer designs

Numerous applications in integrated photonics require more sophisticated architectures that provide enhanced control, multiple functionalities, or specialized spectral responses beyond what single interferometric elements can achieve. The proposed Michelson design can be expanded through two fundamental architectural approaches: cascading and branching.

Cascading involves connecting multiple interferometric stages in series to achieve improved spectral selectivity, enhanced extinction ratios, or more complex transfer functions, and is a common strategy in MZI based filters^44^. Reflector-terminated Mach-Zehnder interferometers, such as the one by Raghi et. al^45^., commonly use loop reflectors to improve the device’s filtering capabilities by providing additional degrees of freedom for spectral engineering while preserving the benefits of the reflective architecture. The basic Michelson structure can be made to serve a similar function by cascading an additional stage of directional coupler after the basic Michelson structure, as shown in Fig. 5c.

Branching, conversely, involves splitting the interferometer arms into multiple parallel paths, enabling simultaneous processing of different optical signals or providing independent control over multiple output channels. This can be seen in optical in-phase/quadrature modulators (IQM), which incorporate Y-branches to separate the signal into multiple sub-arms. This branched configuration is usually achieved with MZI^46–48^, and enables complex modulation schemes, parallel signal processing, or applications requiring simultaneous access to orthogonal phase relationships. A similar arrangement can be achieved with the proposed Michelson and Y-splitters, as shown in Fig. 5d.

These extensions are intended as proof-of-concept demonstrations of the architecture flexibility of the platform, and are in no way an exhaustive list of possibilities. The designs demonstrate the inherent flexibility of the platform while maintaining the advantageous properties of the core Michelson configuration, including the increased phase sensitivity and compact footprint enabled by the reflective geometry. Both devices were fabricated with results presented in Section Fabrication and Characterization.

## Fabrication and characterization

**Fig. 8:**
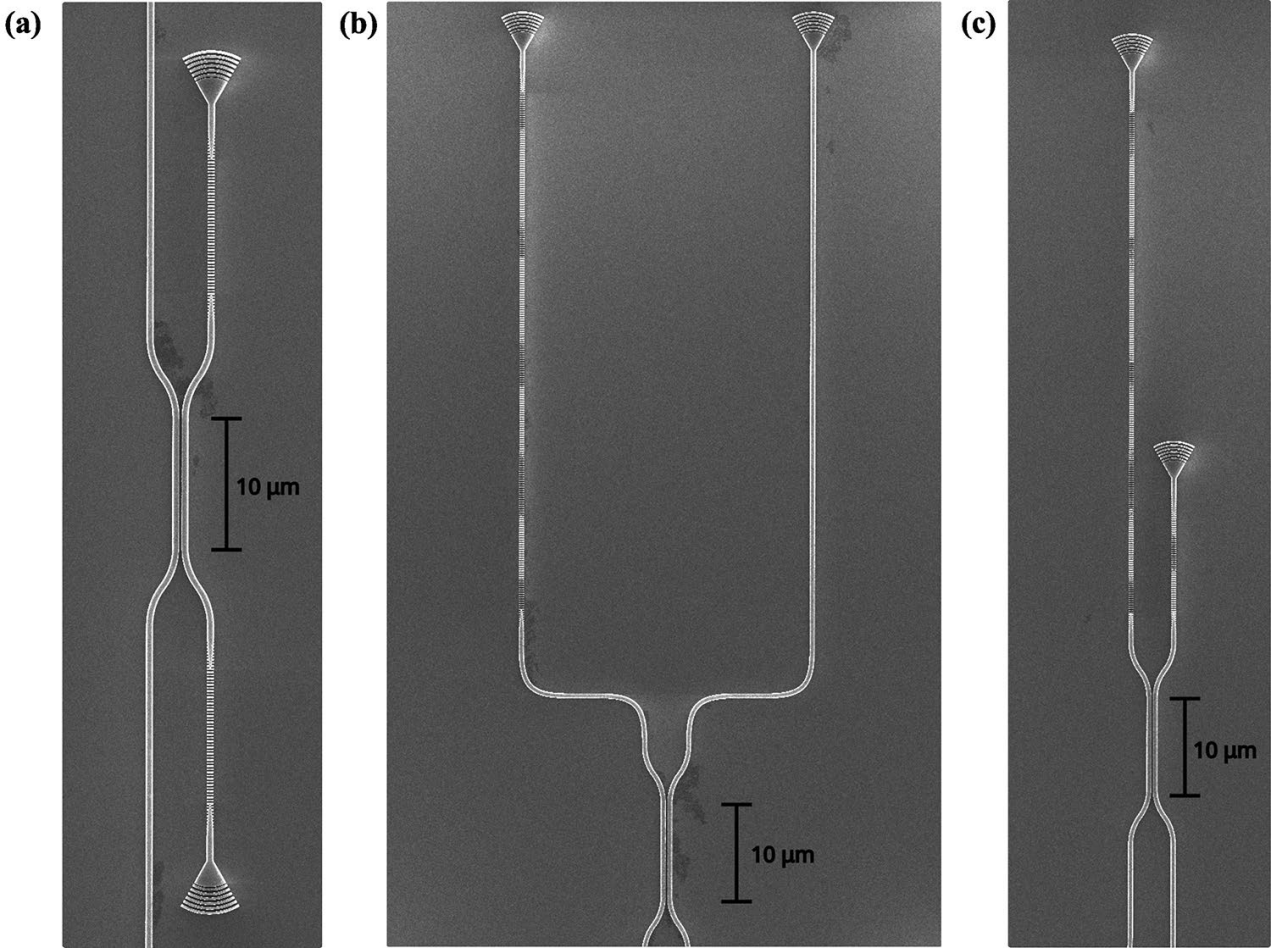
SEM images of fabricated devices. (**a**) is the smaller of the two fabricated michelson interferometers, (**b**) a hybrid michelson with a grated arm and a waveguide arm of equal length, and (**c**) is the michelson of middle $\Delta L$. Images have been digitally altered to increase contrast and brightness.

**Fig. 9:**
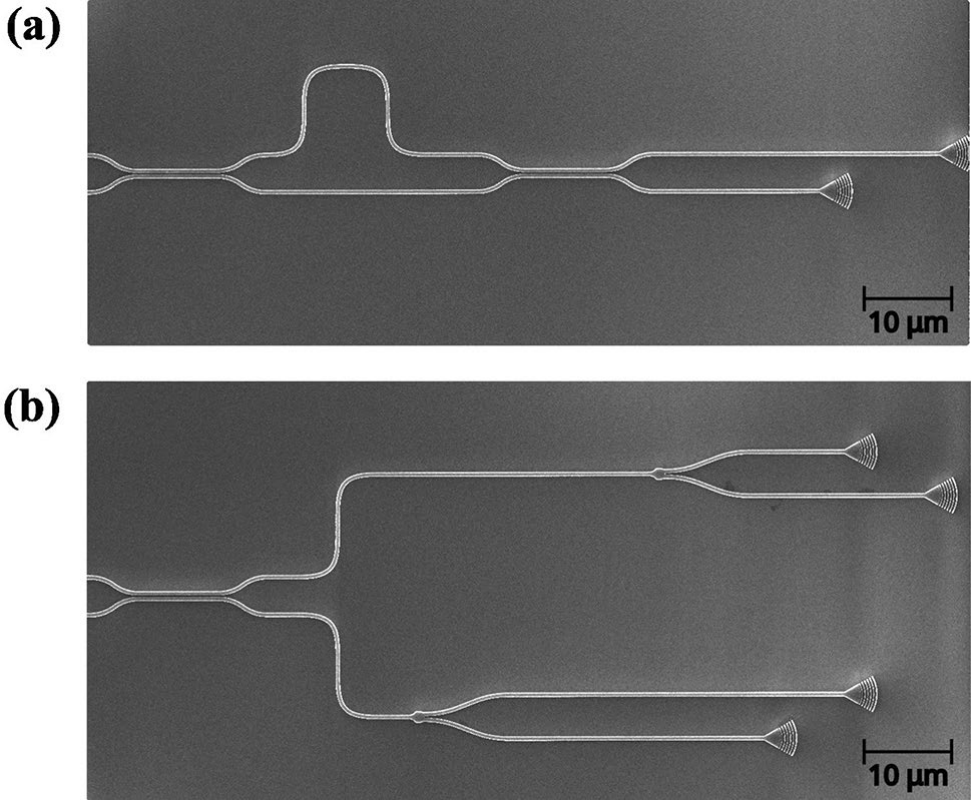
SEM images of the more complex fabricated devices, (**a**) being the reflection terminated MZI and (**b**) being the IQM. Images have been digitally altered to increase contrast and brightness.

**Fig. 10:**
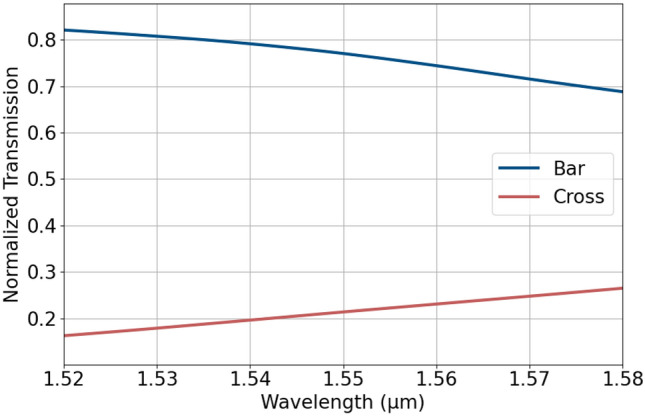
Simulated normalized power transmission for the bar and cross ports of the directional coupler utilized across all interferometer configurations. At the 1.55 $\mu$m central wavelength, the coupler exhibits a splitting ratio of approximately 80:20.

All devices were fabricated using electron beam lithography^49^ on a silicon-on-insulator (SOI) platform with a 220 nm silicon device layer on buried oxide. A single-step full silicon etch was performed using HSQ negative resist, after which the structures were clad with silicon dioxide. This process has relatively high resolution, which is necessary for the fabrication of the subwavelength gratings (SWGs) and compact reflectors. All SWG sections in this work were designed with a period of 240 nm and duty cycle of 0.5. Representative scanning electron microscope (SEM) images of the fabricated devices are shown in Figures 8 and 9, captured prior to silicon dioxide cladding deposition.

The directional coupler integrated into every interferometric architecture in this study is designed to operate with a splitting ratio of approximately 80:20 at the 1.55 $\mu$m central wavelength, as illustrated in the simulated spectral response in Fig. 10. This is achieved using a coupling length of 10 $\mu$m and a 200 nm gap between the 500 nm wide waveguides. Over the range, it varies from a minimum coupling ratio of approximately 86:14 to a maximum of 70:30. The reflector is the one introduced in Section Simulation and Device Modeling.

**Fig. 11:**
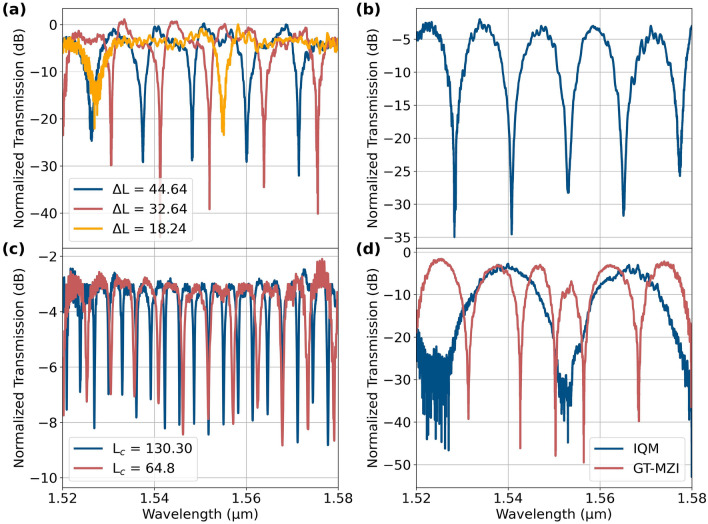
Fabricated device responses over the infrared range of 1.52–1.58 $\mu$m: (**a**) comparison of responses for different lengths of SWG assisted Michelson interferometers. (**b**) Response of hybrid Michelson interferometer with equal length arms but SWG on one and a standard waveguide on the other (**c**) comparison of responses for Fabry-Pérot Interferometers of different cavity lengths. (**d**) Responses for both the GT-MZI and the IQM.

Optical characterization was performed using a tunable laser source spanning 1500–1580 nm. Light was coupled in and out of the chip using surface grating couplers and a polarization-maintaining fiber array with 127 $\mu$m pitch. To ensure the tunable laser source remained stable against any back-propagating signals from the directional couplers, measurements were performed using an HP 81680 A Tunable Laser, which features a built-in optical isolator that provides an output isolation of 50 dB. The measured spectral responses of the fabricated devices are summarized in Fig. 11. While the spectral features show the expected qualitative behavior, absolute wavelength positions exhibit systematic shifts relative to simulations, attributed primarily to fabrication-induced dimensional variations in the waveguide cross-section and grating features. Such shifts are common in high-resolution photonic devices and can be compensated through post-fabrication trimming or pre-corrected in design iterations.

Despite these wavelength offsets, key performance metrics align with theoretical predictions. For the Michelson interferometers shown in Fig. 11a, the free spectral range scales inversely with the path length difference between arms, as expected. Similarly, the Fabry-Pérot interferometer cavity length determines the FSR, with longer cavities exhibiting proportionally more resonances within the measurement window. The measured extinction ratios, fringe visibility, and full spectral range align well with expected behaviors.

**Table 1:** Comparison of fabricated Fabry-Pérot (FP) devices. Both devices feature a directional coupler with $\kappa =0.1$ and SWG sections with DC=0.5. The free spectral range (FSR) and experimental quality factor (Q) are caclulated as an averaged across resolvable resonances within the measured spectra, with Q calculated via Eq. 9. Insertion losses are normalized to a reference straight waveguide.

$\boldsymbol{L_c}$ ($\boldsymbol{\mu }$m)	$\boldsymbol{L_{SWG}}$ ($\boldsymbol{\mu }$m)	Q-Factor	FSR (nm)	Insertion loss (dB)
64.8	28.4	3655	5.354	2.1441
130.3	96.2	6566	3.153	4.2510

**Table 2:** Design Parameters for the reflector terminated IQM and MZI shown in Fig. 9. The IQM values are optimized such that the device displays a single peak at the center of the experimental spectra range at 1.55 $\mu m$, while the MZI was designed to produce several highly visible peaks. SWG sections were not included in these devices.

Device	$\boldsymbol{\Delta L_1}$ ($\boldsymbol{\mu }$m)	$\boldsymbol{\Delta L_2}$ ($\boldsymbol{\mu }$m)	$\boldsymbol{\Delta L_3}$ ($\boldsymbol{\mu }$m)
Reflector terminated IQM	30	10	10
Reflector terminated MZI	16.15	15	-

**Table 3:** Comparison of Michelson based devices fabricated within this study. FSR and ER are calculated as an average over all sufficiently noiseless resonances within the experimental spectra. Insertion losses are normalized to a reference straight waveguide. Note that $\Delta L$ values listed are composed entirely of SWG sections.

Device	ER (dB)	FSR (nm)	Insertion Loss (dB)
Michelson with $\Delta L =18.24\mu m$	32.92	28.160	0.3822
Michelson with $\Delta L =32.64 \mu m$	42.61	11.24	0.9330
Michelson with $\Delta L =44.64 \mu m$	33.39	11.315	1.1451
Hybrid arm Michelson ($L_{SWG}=57.4 \mu m$)	40.21	9.703	0.2193
Reflector terminated MZI	33.39	11.315	0.5768
Reflector terminated IQM	43.68	—	1.1843

## Discussion

### Optical filtering applications

Optical filtering involves the selective transmission or rejection of specific wavelengths from a broadband optical signal, forming the foundation of wavelength division multiplexing systems, spectroscopic instruments, and channel selection in optical networks. The ability to achieve high spectral selectivity, wide free spectral ranges, and low insertion losses within compact integrated devices is crucial for next-generation photonic systems.

The reflective architectures presented in this work offer advantages for optical filtering applications compared to conventional integrated photonic devices. Both the Fabry-Pérot and Michelson interferometer designs can function as optical filters, though in different filtering scenarios due to their distinct spectral characteristics.

Ring resonators are among the most common tools for integrated optical filtering due to their compact footprint and high quality factors. However, the Fabry-Pérot interferometer can achieve comparable filtering performance while offering distinct advantages. Unlike ring resonators, which suffer from increased radiation losses and fabrication challenges when scaled to small radii for large free spectral ranges, the FP design maintains its performance characteristics across different cavity lengths. This flexibility makes FP interferometers particularly suitable for applications requiring wide FSR operation or where precise spectral control is needed.

The multiple round-trip reflections within the FP cavity create sharp, periodic transmission peaks with high extinction ratios between resonances. This makes FP devices useful for wavelength-division multiplexing (WDM) applications, channel dropping, and spectroscopic filtering where high spectral selectivity is required. The Q-factor scales directly with cavity length and mirror reflectivity, enabling optimization for specific filtering requirements. The integration of subwavelength grating sections provides additional degrees of freedom for fine-tuning filter characteristics, with the duty cycle enabling precise effective index control for post-fabrication optimization.

### Active modulation applications

Optical modulation enables the encoding of information onto optical carriers through controlled manipulation of amplitude, phase, or frequency, serving as the cornerstone of high-speed optical communication systems. As data rates continue to scale toward terabit-per-second levels, the demand for power-efficient, compact modulators with high bandwidth and linear response becomes increasingly critical for sustainable photonic networks.

The Michelson interferometer shows potential for optical modulation, offering enhanced sensitivity relative to conventional designs. Its reflective configuration enables efficient active control within a compact footprint, well suited for large-scale photonic integration. The inherent twofold phase sensitivity, resulting from the round-trip propagation in each arm, directly improves modulation efficiency. Consequently, comparable modulation depths can be realized with approximately half the voltage or thermal tuning power required in Mach-Zehnder interferometer (MZI) modulators. While MZIs remain the standard in optical communications owing to their maturity, the reflection based interferometer designs can provide superior power efficiency, which an increasingly important advantage in dense integrated photonic circuits where power and thermal management is a key constraints.

For modulation applications, a 50:50 coupling ratio maximizes trough depth, and introducing deviations DC offsets that limit modulation swing. Subwavelength grating sections further enable precise control of phase, a requirement for coherent systems where phase and amplitude control must be maintained across multiple channels.

### Optical sensing applications

**Fig. 12:**
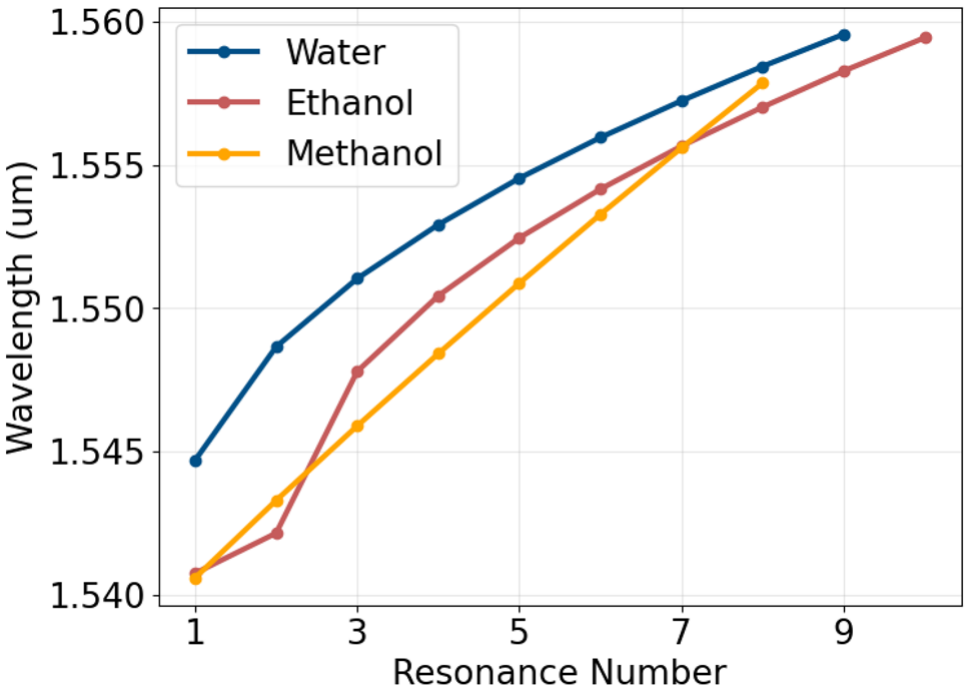
Simulation resonance wavelengths for the fabricated SWG-assisted FP with $L_c = 64.8$ $\mu$m under different mediums between $\lambda = 1.54$–1.56 $\mu$m.

Optical sensing relies on changes in the refractive index of the surrounding medium to detect chemical or biological analytes, with applications ranging from environmental monitoring to biomedical diagnostics^50^. The performance of optical sensors is fundamentally limited by their ability to efficiently couple light into the analyte region while maintaining high sensitivity and spectral resolution within compact device footprints. The reflective interferometric architectures presented in this work offer significant advantages for sensing applications through their enhanced sensitivity and design flexibility. The main performance parameter of optical sensors is the Figure of merit (FOM), defined as:

15 $$\begin{aligned} \text {FOM} = \frac{S}{\text {FWHM}} \end{aligned}$$

where *S* is the wavelength sensitivity and FWHM is the full width at half maximum of the resonance linewidth. Both the Fabry-Pérot and Michelson interferometer configurations can be optimized for sensing. For the Fabry-Pérot interferometer operating as a sensor, when the cavity is exposed to an analyte, changes in the surrounding refractive index $n_{\text {med}}$ modify the effective index through evanescent field coupling. The wavelength sensitivity of the FP sensor is given by:

16 $$\begin{aligned} S_{\text {FP}} = \frac{\partial \lambda _{\text {res}}}{\partial n_{\text {med}}} = \frac{\lambda _{\text {res}}}{n_{\text {eff}}} \cdot \frac{\partial n_{\text {eff}}}{\partial n_{\text {med}}} = \frac{\lambda _{\text {res}}}{n_{\text {eff}}} \cdot S_{\text {wg}} \end{aligned}$$

which comes from Eq. 4. Combining this with eq9, it follows that the FOM of the FP sensor becomes:

17 $$\begin{aligned} \text {FOM}_{\text {FP}} = \frac{S_{\text {FP}}}{\text {FWHM}} = \frac{\pi L_c S_{\text {wg}}}{1-R} \end{aligned}$$

This expression shows that the FP sensor FOM scales directly with cavity length and waveguide sensitivity, while being inversely proportional to mirror losses. Given the low losses of the circular-Bragg reflector, a high Q factor is achievable, enabling extremely narrow linewidths, providing a better resolution/footprint tradeoff compared to other sensing architectures. Figure 12 demonstrates the use of a SWG assisted SOI FP.

For the Michelson interferometer operating as a sensor, the wavelength sensitivity can be derived as:

18 $$\begin{aligned} S_{\text {MI}} = \frac{\partial \lambda _{\text {res}}}{\partial n_{\text {med}}} = \frac{\lambda _{\text {res}}}{n_{\text {eff,sens}}L_{\text {sens}} - n_{\text {eff,ref}}L_{\text {ref}}} \cdot 2L_{\text {sens}} \cdot S_{\text {wg}} \end{aligned}$$

where $S_{\text {wg}} = \partial n_{\text {eff}}/\partial n_{\text {med}}$ is the waveguide sensitivity parameter that quantifies how changes in the medium refractive index $n_{\text {med}}$ affect the effective index of the guided mode.

The choice between FP and Michelson configurations for sensing applications depends on the specific requirements. The FP sensor is better in applications requiring ultra-high resolution due to its narrow linewidths and high quality factors, making it ideal for detecting minute refractive index changes or for spectroscopic applications. The Michelson sensor, conversely, offers superior design flexibility through independent control of sensing and reference arm parameters, enabling optimization for specific sensitivity ranges. Furthermore it has an inherently higher temperature robustness compared to the resonator. Tables 5 and 4 compare the sensitivity of the devices to comparable devices in the literature, with MI compared to MZI based devices and the FP compared to resonators, each calculated analytically using the formulas derived in this section.

**Table 4:** An overview of of micro-ring and photonic resonator sensors is presented, highlighting their sensing applications, sensitivities, and device dimensions in comparison with the results of this work. Results for this work are calculated using Eq.16 and simulation results for waveguide sensitivities from Section Simulation and Device Modeling. It is noted that variations in cavity length do not alter sensitivity, as the multiple light circulations within the resonator means that the effective light-matter interaction remains independent of the physical cavity length.

Device	Application	Sensitivity	Radius/Length
Si SOI Slot MRR^51^	RI sensor	545 nm/RIU	R = 10 $\mu$m
Si Photonic MRR^52^	RI sensor	400 nm/RIU	R = 15 $\mu$m
PDMS Slot MRR^53^	Temperature	151 pm/$^\circ$C	R = 10 $\mu$m
SiN MRR^54^	Biosensing	100 nm/RIU	R = 30 $\mu$m
SOI Resonator^55^	Chemical, RI	250 nm/RIU	R = 10 $\mu$m
SiN CROW^56^	Biosensing	384 nm/RIU	R = 10 $\mu$m
SWG MRR^57^	RI Sensor	440.5 nm/RIU	R = 10 $\mu$m
SWG Assisted Fabry-Pérot (This work)	RI sensor	596 nm/RIU	L = 64.8 $\mu$m
SWG Assisted Fabry-Pérot (This work)	RI sensor	596 nm/RIU	L = 130.3 $\mu$m

**Table 5:** Summary of reported MZI sensors with their sensing type, sensitivities, and sensing lengths compared to results in this work. Literature values originally reported in phase sensitivity were converted to wavelength sensitivity for direct comparison. Results for this work are calculated using Eq.18 and simulation results from Section Simulation and Device Modeling.

Device	Application	Sensitivity (nm/RIU)	Sensing Length
WCSW Silica MZI^58^	RI sensor	$\sim$407$^*$	15 mm
Slot SiN MZI^59^	Biosensor	$\sim$177$^\dagger$	7 mm
Photonic Crystal MZI^60^	Biosensor	103	16 $\mu$m
Si Gas MZI^61^	Gas sensor	1,458	10 mm
SWG Assisted Michelson (This work)	RI sensor	490.3	18.24 $\mu$m
SWG Assisted Michelson (This work)	RI sensor	273.9	32.64 $\mu$m
SWG Assisted Michelson (This work)	RI sensor	200.8	44.64 $\mu$m

$^*$Calculated from the reported intrinsic waveguide sensitivity ($S_{wg} = 0.94$) at $\lambda = 632.8$ nm. $^\dagger$Calculated from the reported phase sensitivity ($1864\pi$/RIU) at $\lambda = 1550$ nm.

The incorporation of subwavelength grating sections in the sensing arms again enhances the performance of both interferometric architectures. Owing to their periodically open structure, SWG waveguides provide stronger evanescent field coupling and a larger effective surface area for analyte interaction than conventional strip waveguides. Combined with the doubled optical path length inherent to the reflective design, this leads to improved sensitivity over traditional platforms. Moreover, the ability to optimize the duty cycle across different sections offers better opportunities to improve device selectivity, as evidenced by Fig. 2.

**Fig. 13:**
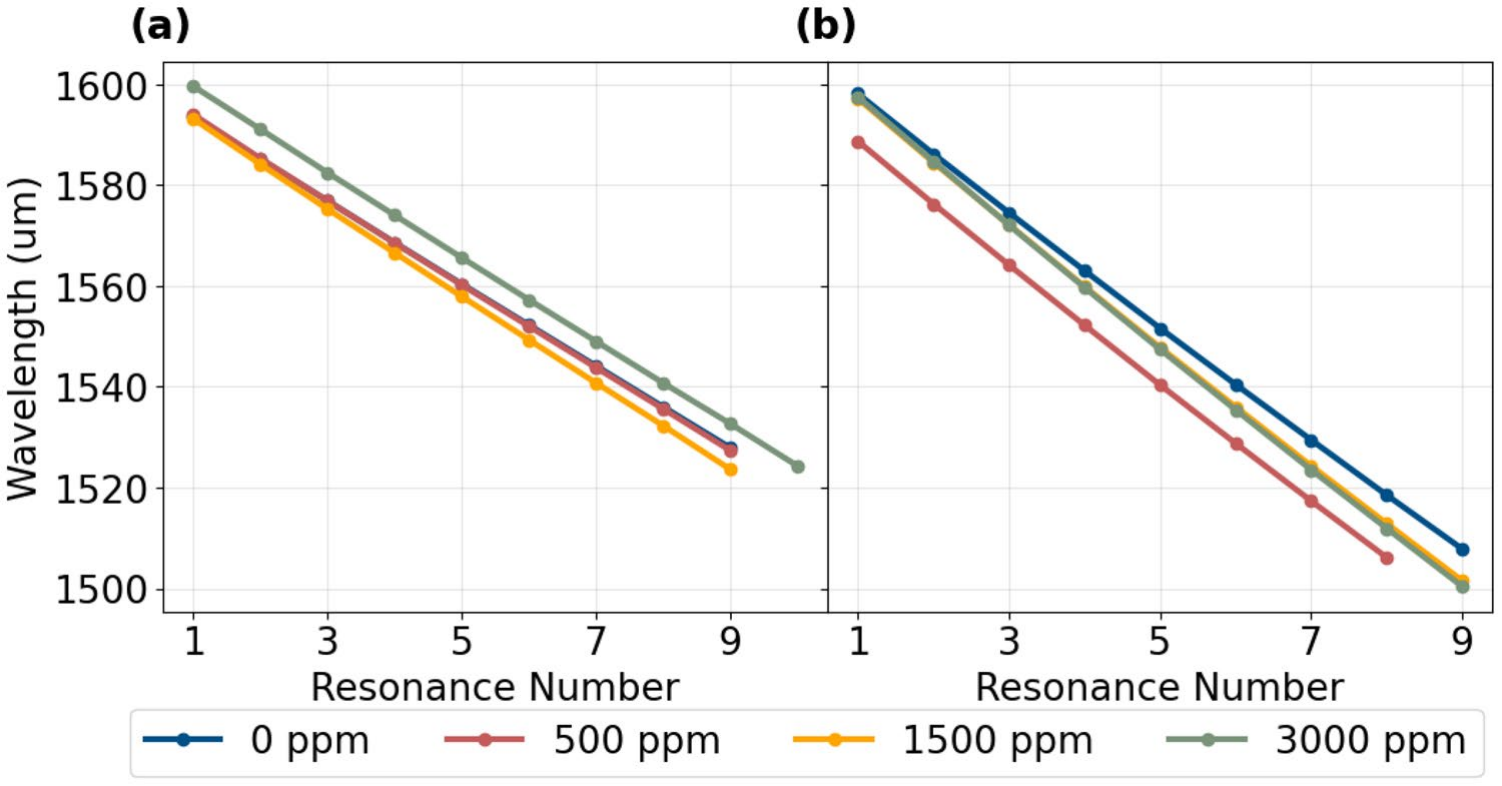
Simulation of resonance wavelengths under varying concentrations of $\mathrm {NO_2}$ for (**a**) a michelson with arms of equal length =50 $\mu$m with a silicon waveguide and a P3OT waveguide (**b**) a Fabry-Pérot resonator with a cavity length of 50 $\mu$m composed of P3OT.

Another potential way to utilize the design is to replace the arms or cavity waveguides with polymers sensitive to target chemicals^62^. This approach, called Silicon-Organic Hybrid (SOH) photonics, requires an additional polymer cladding deposition and patterning step. It’s usually achieved using adiabatic coupling structures, such as long tapers that gradually end in a sharp tip, so that the optical field gradually leaks out of the silicon into the surrounding polymer cladding. As a result, the mode’s effective index smoothly transitions from being dominated by the silicon core to being guided by the lower-index polymer waveguide structure built above it. This slow, gradual transition keeps the light in the fundamental mode, minimizing loss and back-reflection. Several polymers have been shown to have use in the context of sensing, such as P3OT being sensitive to nitrogen dioxide ($\mathrm {NO_2}$)^63^ and PMMA responding to methanol and ethanol^64^. As a proof of concept, Fig. 13 demonstrates how the response of a michelson and Fabry Pérot integrated with P3OT arms changes as a function of ($\mathrm {NO_2}$) concentration.

## Conclusion

In this work, a series of Bragg-based interferometric devices were designed and experimentally characterized on the silicon-on-insulator platform. Compact architectures incorporating circular Bragg grating reflectors were implemented in four configurations: Fabry-Pérot interferometers, Michelson interferometers, grating-terminated Mach-Zehnder interferometers, and in-phase/quadrature modulators. The use of circular Bragg reflectors enabled substantial footprint reduction compared with conventional linear designs, while preserving broadband, high-reflectivity performance.

Experimental characterization exhibited qualitative agreement with numerical simulations. The Fabry-Pérot structures achieved quality factors exceeding 6,500, whereas the Michelson-based devices demonstrated extinction ratios up to 43 dB. The integration of subwavelength grating (SWG) sections provided additional control over the effective index and enhanced the evanescent field overlap, thereby improving the tunability and sensitivity of the devices for potential sensing and modulation applications.

The reflective Michelson configuration inherently offers a twofold increase in phase sensitivity relative to conventional Mach-Zehnder interferometers, enabling higher modulation efficiency and improved phase resolution. Furthermore, the demonstrated cascading and branching extensions highlight the architectural versatility of the platform, facilitating the realization of advanced functionalities such as multi-channel filtering and modulation within compact footprints.

Overall, the presented results show potential of the architectures as a versatile and scalable foundation for future compact interferometric devices in integrated photonics. With optimization, these structures can be designed for diverse applications including high-resolution sensing, optical signal processing, and efficient on-chip modulation.

## Data Availability

The datasets used and/or analyzed during the current study are available from the corresponding author on reasonable request.

## References

[1] Hochberg, M. et al. Silicon photonics: The next fabless semiconductor industry. *IEEE Solid-State Circuits Mag.* **5**, 48–58 (2013).

[2] Hochberg, M. & Baehr-Jones, T. Towards fabless silicon photonics. *Nat. Photonics* **4**, 492–494 (2010).

[3] Soref, R. The past, present, and future of silicon photonics. *IEEE J. Sel. Top. Quantum Electron.* **12**, 1678–1687. 10.1109/JSTQE.2006.883151 (2006).

[4] Reed, G. T., Mashanovich, G., Gardes, F. Y. & Thomson, D. Silicon optical modulators. *Nat. Photonics* **4**, 518–526 (2010).

[5] Chrostowski, L. & Hochberg, M. *Silicon photonics design: from devices to systems* (Cambridge University Press, 2015).

[6] Bogaerts, W. et al. Silicon microring resonators. *Laser Photonics Rev.* **6**, 47–73 (2012).

[7] Halir, R. et al. Subwavelength-grating metamaterial structures for silicon photonic devices. *Proc. IEEE* **106**, 2144–2157. 10.1109/JPROC.2018.2851614 (2018).

[8] Cheben, P., Halir, R., Schmid, J. H., Atwater, H. A. & Smith, D. R. Subwavelength integrated photonics. *Nature* **560**, 565–572 (2018). 30158604 10.1038/s41586-018-0421-7

[9] Li, C. et al. Subwavelength silicon photonics for on-chip mode-manipulation. *PhotoniX* **2**, 11. 10.1186/s43074-021-00032-2 (2021).

[10] Wangüemert-Pérez, J. G. et al. Subwavelength structures for silicon photonics biosensing. *Opt. Laser Technol.* **109**, 437–448, 10.1016/j.optlastec.2018.07.071.

[11] Meng, Y. et al. Optical meta-waveguides for integrated photonics and beyond. *Light Sci. Appl.* **10**, 235 (2021). 34811345 10.1038/s41377-021-00655-x PMC8608813

[12] Saber, M. G. et al. Transversely coupled Fabry–Perot resonators with Bragg grating reflectors. *Opt. Lett.* **43**, 13. 10.1364/OL.43.000013 (2018). 29328225 10.1364/OL.43.000013

[13] Ansys, Inc. Optical simulation and design software | ansys optics (2025).

[14] Gehring, H. & contributors. Femwell: An open-source finite-element modeling tool for integrated photonics. https://github.com/helgegehring/femwell.

[15] Palik, E. D. *Handbook of Optical Constants of Solids* (Academic Press, 1985).

[16] Ortega-Moñux, A. et al. Disorder effects in subwavelength grating metamaterial waveguides. *Opt. Express.* **25**, 12222–12236 (2017). 28786581 10.1364/OE.25.012222

[17] Bock, P. J. et al. Subwavelength grating periodic structures in silicon-on-insulator: A new type of microphotonic waveguide. *Opt. Express.* **18**, 20251–20262 (2010). 20940916 10.1364/OE.18.020251

[18] Wang, Y., Gao, S., Wang, K., Li, H. & Skafidas, E. Ultra-broadband, compact, and high-reflectivity circular Bragg grating mirror based on 220 nm silicon-on-insulator platform. *Opt. Express* **25**, 6653–6663. 10.1364/OE.25.006653 (2017). 28381010 10.1364/OE.25.006653

[19] Fernández-Hinestrosa, A. et al. Nanophotonic bragg grating assisted mach-zehnder interferometers for o-band add-drop filters. *Sci. Rep.* **14**, 18492 (2024). 39122818 10.1038/s41598-024-69042-x PMC11315689

[20] Shao, H., Wang, Y., Yang, G. & Sang, T. Topological transport in heterostructure of valley photonic crystals. *Opt. Express* **31**, 32393–32403 (2023). 37859044 10.1364/OE.494644

[21] Mizumoto, T., Baets, R. & Bowers, J. E. Optical nonreciprocal devices for silicon photonics using wafer-bonded magneto-optical garnet materials. *MRS Bulletin* **43**, 419–424 (2018).

[22] Born, M. & Wolf, E. *Principles of Optics: Electromagnetic Theory of Propagation, Interference and Diffraction of Light* 7 edn. (Cambridge University Press, 1999).

[23] Hariharan, P. *Optical Interferometry* 2 edn. (Academic Press, 2003).

[24] Pruessner, M. W., Stievater, T. H. & Rabinovich, W. S. Integrated waveguide fabry-perot microcavities with silicon/air bragg mirrors. *Opt. Lett.* **32**, 533–535 (2007). 17392912 10.1364/ol.32.000533

[25] Wang, Z., Xu, X., Fan, D., Wang, Y. & Chen, R. High quality factor subwavelength grating waveguide micro-ring resonator based on trapezoidal silicon pillars. *Opt. Lett.* **41**, 3375–3378. 10.1364/OL.41.003375 (2016). 27420539 10.1364/OL.41.003375

[26] Sun, X. et al. Tunable silicon comb filters based on fabry-perot resonators formed by sagnac loop mirrors. In *2013 Optical Fiber Communication Conference and Exposition and the National Fiber Optic Engineers Conference (OFC/NFOEC)*, 1–3 (2013).

[27] Liu, Q. *et al.* On-chip Bragg grating waveguides and Fabry-Perot resonators for long-wave infrared operation up to . *Opt. Express* **26**, 34366, 10.1364/OE.26.034366. 10.1364/OE.26.034366 30650859

[28] Park, Y. J. et al. Fully tunable fabry-pérot cavity based on MEMS sagnac loop reflector with ultra-low static power consumption. *Microsyst. Nanoeng.* **10**, 1–11. 10.1038/s41378-024-00728-y (2024). 39209803 10.1038/s41378-024-00728-y PMC11362568

[29] Pruessner, M. W., Stievater, T. H. & Rabinovich, W. S. Integrated waveguide fabry-perot microcavities with silicon/air bragg mirrors. *Opt. Lett.* **32**, 533–535. 10.1364/OL.32.000533 (2007). 17392912 10.1364/ol.32.000533

[30] Xu, H., Qin, Y., Hu, G. & Tsang, H. K. Million-Q integrated fabry-perot cavity using ultralow-loss multimode retroreflectors. *Photonics Res.* **10**, 2549–2559. 10.1364/PRJ.470644 (2022).

[31] Vivien, L. & Pavesi, L. (eds) *Handbook of Silicon Photonics* (CRC Press, 2016).

[32] Wang, Z., Xu, X., Fan, D., Wang, Y. & Chen, R. T. High quality factor subwavelength grating waveguide micro-ring resonator based on trapezoidal silicon pillars. *Opt. Lett.* **41**, 3375–3378 (2016). 27420539 10.1364/OL.41.003375

[33] Tang, R. et al. Waveguide-multiplexed photonic matrix–vector multiplication processor using multiport photodetectors. *Optica* **12**, 812–820. 10.1364/OPTICA.552023 (2025).

[34] Zhou, H. et al. Photonic matrix multiplication lights up photonic accelerator and beyond. *Light Sci. Appl.* **11**, 30. 10.1038/s41377-022-00717-8 (2022). 35115497 10.1038/s41377-022-00717-8 PMC8814250

[35] Peserico, N., Shastri, B. J. & Sorger, V. J. Integrated Photonic Tensor Processing Unit for a matrix multiply: A review. *J. Lightwave Technol.* **41**, 3704–3716. 10.1109/JLT.2023.3269957 (2023).

[36] Rafiee, E. & Emami, F. Investigating the effects of structural parameters on the optical characteristics of add-drop filters. *Optik* **127**, 1690–1694. 10.1016/j.ijleo.2015.10.232 (2016).

[37] Yan, H., Feng, X., Zhang, D. & Huang, Y. Integrated optical add-drop multiplexer based on a compact parent-sub microring-resonator structure. *Opt. Commun.* **289**, 53–59. 10.1016/j.optcom.2012.09.059 (2013).

[38] Malak, M. Beyond interferometers based on silicon-air Bragg reflectors: Toward on-chip optical microinstruments–A review. *IEEE J. Sel. Top. Quantum Electron.* **21**, 49–60. 10.1109/JSTQE.2014.2372052 (2015).

[39] Gerguis, J. O. & Qi, M. Linear and passive silicon-on-insulator refractive index sensor utilizing Bragg grating-assisted Michelson interferometer. *Opt. Express.* **33**, 9102–9116. 10.1364/OE.537931 (2025). 40798591 10.1364/OE.537931

[40] Ghoname, A. O., Hassanien, A. E., Chow, E., Goddard, L. L. & Gong, S. Highly linear lithium niobate Michelson interferometer modulators assisted by spiral Bragg grating reflectors. *Opt. Express* **30**, 40666. 10.1364/OE.472673 (2022). 36298997 10.1364/OE.472673

[41] Patel, D. et al. High-speed compact silicon photonic Michelson interferometric modulator. *Opt. Express* **22**, 26788. 10.1364/OE.22.026788 (2014). 25401826 10.1364/OE.22.026788

[42] Xu, M. et al. Michelson interferometer modulator based on hybrid silicon and lithium niobate platform. *APL Photonics*10.1063/1.5115136 (2019).

[43] Lu, Z., Murray, K., Jayatilleka, H. & Chrostowski, L. Michelson Interferometer Thermo-Optic Switch on SOI With a 50- Power Consumption. *IEEE Photonics Technol. Lett.* **27**, 2319–2322, 10.1109/LPT.2015.2462341 (2015).

[44] Jinguji, K. & Oguma, M. Optical half-band filters. *J. Light. Technol.* **18**, 252 (2000).

[45] El Shamy, R. S., Afifi, A. E., Badr, M. M. & Swillam, M. A. Modelling, characterization, and applications of silicon on insulator loop terminated asymmetric Mach Zehnder interferometer. *Sci. Rep.* **12**, 3598, 10.1038/s41598-022-07449-0 (2022). 10.1038/s41598-022-07449-0 PMC8897405 35246570

[46] Ogawa, K. Increase in modulation speed of silicon photonics modulator with quantum-well slab wings: New insights from a numerical study. *Photonics* **11**, 10.3390/photonics11060535 (2024).

[47] Hasan, G. M., Hasan, M. & Hall, T. J. Performance analysis of a multi-function mach-zehnder interferometer based photonic architecture on soi acting as a frequency shifter. *Photonics* **8**, 10.3390/photonics8120561 (2021).

[48] Sepehrian, H., Lin, J., Rusch, L. A. & Shi, W. Silicon photonic iq modulators for 400 gb/s and beyond. *J. Light. Technol.* **37**, 3078–3086 (2019).

[49] Chrostowski, L. SiEPIC EBeam PDK & Library, for SiEPIC-Tools and KLayout: lukasc-ubc/SiEPIC_EBeam_PDK (2019).

[50] Passaro, V. M. et al. Recent advances in integrated photonic sensors. *Sensors (Basel)* **12**, 15558–15598 (2012). 23202223 10.3390/s121115558 PMC3522976

[51] Schweikert, C. et al. Thermally robust silicon integrated ring resonators with postprocessing sensitivity enhancement for biosensing. *J. Light. Technol.* **43**, 4819–4825 (2025) .

[52] Fallahi, V., Kordrostami, Z. & Hosseini, M. Sensitivity and quality factor improvement of photonic crystal sensors by geometrical optimization of waveguides and micro-ring resonators combination. *Sci. Rep.* **14**, 2001 (2024). 38263207 10.1038/s41598-024-52363-2 PMC10805923

[53] Chu, X. et al. Design of ultra-high sensitivity slot micro-ring resonator acoustic sensor. *Fiber Integr. Opt.* **41**, 83–95 (2022).

[54] Bryan, M. R., Butt, J. N., Bucukovski, J. & Miller, B. L. Biosensing with silicon nitride microring resonators integrated with an on-chip filter bank spectrometer. *ACS Sens.* **8**, 739–747 (2023). 36787432 10.1021/acssensors.2c02276 PMC9972465

[55] Zhang, W., Zhu, H. & Lee, J.-Y. Piezoresistive transduction in a double-ended tuning fork SOI MEMS resonator for enhanced linear electrical performance. *IEEE Trans. Electron Devices* **62**, 1596–1602 (2015).

[56] Xu, D.-X. et al. Frontiers original research in materials published: 27 april 2015 . *Photonic Integration and Photonics-Electronics Convergence on Silicon Platform* 32 10.3389/fmats.2015.00034 (2015).

[57] Yan, H. et al. Unique surface sensing property and enhanced sensitivity in microring resonator biosensors based on subwavelength grating waveguides. *Opt. Express* **24**, 29724–29733 (2016). 28059356 10.1364/OE.24.029724 PMC5234505

[58] Zhao, C., Xu, L. & Liu, L. Ultrahigh Sensitivity Mach–Zehnder Interferometer Sensor Based on a Weak One-Dimensional Field Confinement Silica Waveguide. *Sensors* **21**, 6600, 10.3390/s21196600 (2021). 10.3390/s21196600 PMC8512376 34640918

[59] Liu, Q. et al. Highly sensitive Mach-Zehnder interferometer biosensor based on silicon nitride slot waveguide. *Sens. Actuators B Chem.* **188**, 681–688 (2013).

[60] Qin, K., Hu, S., Retterer, S. T., Kravchenko, I. I. & Weiss, S. M. Slow light Mach-Zehnder interferometer as label-free biosensor with scalable sensitivity. *Opt. Lett.* **41**, 753–756 (2016). 26872180 10.1364/OL.41.000753

[61] Taha, A. M., Yousuf, S., Dahlem, M. S. & Viegas, J. Highly-sensitive unbalanced MZI gas sensor assisted with a temperature-reference ring resonator. *IEEE Photonics J.* **14**, 1–9 (2022).

[62] Swillam, S. M. et al. Polymer-based michelson and fabry-perot interferometers for optical gas sensing. In *Integrated Optics: Devices, Materials, and Technologies XXX*, vol. 13900, 217–219 (SPIE, 2026).

[63] Solís, J. C., De la Rosa, E. & Cabrera, E. P. Absorption and refractive index changes of poly (3-octylthiophene) under NO_2_ gas exposure. *Opt. Mater.* **29**, 167–172 (2006).

[64] Krämmer, S. et al. Electrospun polymer fiber lasers for applications in vapor sensing. *Adv. Opt. Mater.* **5**, 1700248 (2017).

